# Influence of Immobilization, Stretching, and Activity on the Morphological Properties of Spastic Gastrocnemius Muscles

**DOI:** 10.3390/children13030414

**Published:** 2026-03-18

**Authors:** Andreas Habersack, Annika Kruse, Bernhard Guggenberger, Nina Mosser, Markus Tilp, Martin Svehlik

**Affiliations:** 1 Department of Human Movement Science, Sport and Health, University of Graz, Aigner-Rollett-Allee 39, 8010 Graz, Austria; andreas.habersack@uni-graz.at (A.H.); nina.mosser@uni-graz.at (N.M.); markus.tilp@uni-graz.at (M.T.); 2 Department of Orthopaedics and Trauma, Medical University of Graz, Auenbruggerplatz 34, 8036 Graz, Austria; bernhard.guggenberger2@fh-joanneum.at (B.G.); martin.svehlik@medunigraz.at (M.S.); 3 Institute of Physiotherapy, JOANNEUM University of Applied Sciences, Alte Poststraße 149, 8020 Graz, Austria

**Keywords:** cerebral palsy, orthotic treatment, equinus, muscle, Achilles tendon

## Abstract

**Highlights:**

**What are the main findings?**

- No muscle atrophy or fascicle shortening after moderate-duration orthotic treatments.
- Structural adaptations were only observed in the Achilles tendon.

**What are the implications of the main findings?**

- Orthotic treatment does not seem to result in negative muscle adaptations when performed for no longer than 3 months.
- Orthotic-induced stretching primarily affects the tendon rather than the spastic muscle.

**Abstract:**

**Background/Objectives:** Children with cerebral palsy (CP) often develop altered muscle architecture and calf muscle contractures. Orthotic immobilization aims to provide prolonged stretch to lengthen the muscle belly and muscle–tendon unit (MTU), but immobilization may also cause atrophy. This study investigated whether immobilization combined with periods of daily muscle activation has a different effect on calf muscle properties than continuous immobilization alone. **Methods**: Fourteen children with CP and equinus deformity (mean age: 9.9 ± 3.0 years; GMFCS Level I: 10, II: 4) were enrolled in a 12-week randomized controlled trial. Participants were allocated to one of two groups: continuous immobilization (23 h per day) with a dynamic ankle–foot orthosis (AFO), or a combined regimen consisting of immobilization (14 h) and a daily activity phase (10 h). Gastrocnemius medialis (GM) MTU properties, including muscle belly and Achilles tendon (AT) length, fascicle length, and muscle volume, among others, were assessed four times using three-dimensional (3D) freehand ultrasound. **Results**: Significant within-group increases in MTU and AT lengths were observed over time at both a 90° ankle position (*p* < 0.01) and a more dorsiflexed ankle position (4 Nm applied torque, *p* < 0.01). However, no significant group × time interactions were observed for any parameter. **Conclusions**: Contrary to our hypothesis, combining activity and immobilization did not confer additional benefits. Nevertheless, shorter orthosis-wearing time had the same effect on the MTU and could lead to improved compliance with orthosis treatment in CP. Larger trials are needed to support our findings.

## 1. Introduction

Cerebral palsy (CP) is an early-onset lifelong neurodevelopmental condition, with affected individuals presenting with limitations in activity due to impaired development of movement and posture resulting from dysplasia of or injury to the fetal or infant brain [1]. Spasticity (i.e., the dynamic resistance to passive movement of a joint) is the most common motor category of individuals with CP [1,2,3]. In children with CP, spastic muscles do not grow in proportion to the bones and exhibit dynamic tightness, which often leads to fixed contractures as they age [4,5,6,7]. The abnormal muscle activity places excessive forces on the growing skeleton, contributing to secondary bony deformities and joint instability [8]. Besides functional changes, CP also alters the architecture of affected muscles [8].

Consistent evidence shows that spastic CP leads to reduced muscle size, including decreases in muscle volume, cross-sectional area, muscle thickness, and muscle belly length when compared to typically developed muscles [9]. Muscle fascicle length is a key determinant of muscle excursion; shorter fascicles limit the range of motion through which muscles can generate force and power [10]. According to Mathewson and Lieber (2015) [8], shorter fascicles in children with CP result in greater relative excursions during the same absolute length change in everyday movements compared to healthy individuals. This leads to sarcomere excursions that cover nearly the entire descending limb of the sarcomere force-length relationship, substantially reducing the active force potential in these children [11,12,13]. The alterations in muscle architecture have important clinical implications, particularly for the development and management of muscle contractures in this population.

A common intervention for children with CP is the treatment of dynamic calf muscle contractures that often result in equinus gait [14]. Spasticity in the calf muscles and equinus gait in children with CP are typically treated with prolonged muscle stretching, casting, ankle–foot orthoses (AFOs), botulinum toxin injections, and/or orthopedic surgery [15]. However, while the functional benefits of orthoses are well established, their impact on the mechano-morphological properties of spastic muscles remains poorly understood [16,17,18].

To date, only one study by Hösl et al. (2015) [19] has investigated the effects of orthoses on the gastrocnemius medialis (GM) muscle morphology in children with CP. After 16 weeks of AFO use, the authors found a 7% reduction in muscle belly length, a 14% reduction in fascicle length, and a 32% decrease in muscle extensibility. At the same time, children walked faster and improved foot lift in the swing phase [19]. These results suggest that orthoses may evoke unfavorable changes in GM muscle properties, potentially leading to muscle atrophy. Although Hösl et al. (2015) [19] provided preliminary insights into the effects of immobilization on spastic muscles, their study does not offer a complete understanding of the structural adaptations underlying the observed functional improvements.

Furthermore, the duration of immobilization is an important factor in orthotic treatment. Individuals with CP typically wear orthoses during both the day and the night. However, prolonged immobilization is known to cause disuse atrophy, characterized by reduced muscle size, strength, myofiber area, and increased muscle protein breakdown [20,21]. Therefore, the optimal orthotic treatment regimen should be long enough to achieve beneficial changes but as short as possible to prevent muscle atrophy. However, there is no information yet on the impact of AFO wearing time on muscle properties and function.

The proposed prospective, randomized controlled study aimed to investigate the effects of orthotic treatment on the spastic GM muscle in children and adolescents with CP. We compared two different orthotic treatment regimens comprising a different AFO approach and wearing time: the standard regimen involved the use of an AFO during both day and night (for 23 h per day; Immobilization Group—IG). For the second novel approach, an innovative orthotic design was used that allowed immobilization (for 14 h per day) and plantarflexor activity (for 10 h per day) (Immobilization/Activity Group—IAG).

We hypothesized that, compared to the IG, the IAG would experience increased GM muscle fascicle lengths and less muscle atrophy following the intervention.

## 2. Materials and Methods

### 2.1. Study Design

The present study was prospectively registered at ClinicalTrials.gov (NCT05269745). The study protocol was approved by the Ethics Committee of the Medical University of Graz (approval number 32-115 ex 19/20), and written consent was obtained from the parents or caregivers in advance.

### 2.2. Participants

A previous study on the effects of orthotic treatment reported a significant decrease (−11.2%) in fascicle length over 17 participants after 16 ± 4 weeks [19]. This was related to a medium effect size of 0.6 [22]. The reported change is clinically relevant, as it would reduce the fascicular contraction velocity by the same extent. In this study, we also chose GM muscle fascicle length as the primary outcome measure. We expected a clinically relevant decrease in the IG but not in the alternative IAG. Assuming a medium effect (partial eta square of 0.1 based on the results from [19], an alpha global level of significance of 0.05, a power of 0.8, and an estimated correlation among repeated measures of 0.7, we calculated a necessary total sample size of 14 subjects, i.e., 7 per group, using G*Power Version 3.1 [23].

The participants (Table 1) were recruited from the orthopedic department of the local University hospital.

All the children were aged 5–15 years, ambulatory, and able to accept and follow verbal instructions. They had limited ankle joint range of motion (i.e., maximum dorsiflexion ≤ 5° with the knees extended) as determined during a clinical examination by a physician. All children were able to walk at least short distances (approx. 30 m) without any walking aids (GMFCS I-III). None of the 14 participants had undergone surgery in their lifetimes, and no one had received a Botulinum toxin A injection within 12 months prior to the measurements. Exclusion criteria were any form of spastic CP or oral antispastic medication or muscle-relaxation medication. . Inclusion criteria were defined to ensure a homogeneous sample with comparable neuromuscular and orthopedic characteristics, while exclusion criteria were applied to minimize confounding effects from prior surgery, acute inflammation, or severe comorbidities. The explicit specification of these criteria enhances reproducibility and reduces potential selection bias, ensuring that observed effects can be attributed to the intervention rather than extraneous variables. The selective voluntary motor control was assessed by use of the “Selective Control Assessment of the Lower Extremity” (SCALE, [24]). Participants were randomized to IG or IAG stratified by Gross Motor Function Classification System level (I + II vs. III) and age (5–10 years vs. 11–15 years). Randomization was performed using a computer-generated stratified block procedure. Randomization was conducted by the principal investigator and the investigator performing the assessments. Group assignments were concealed in sealed opaque envelopes, with no access for other study personnel. Finally, due to limited personnel resources, blinding to group allocation was not feasible in this study.

### 2.3. Intervention Protocol

**Figure 1:**
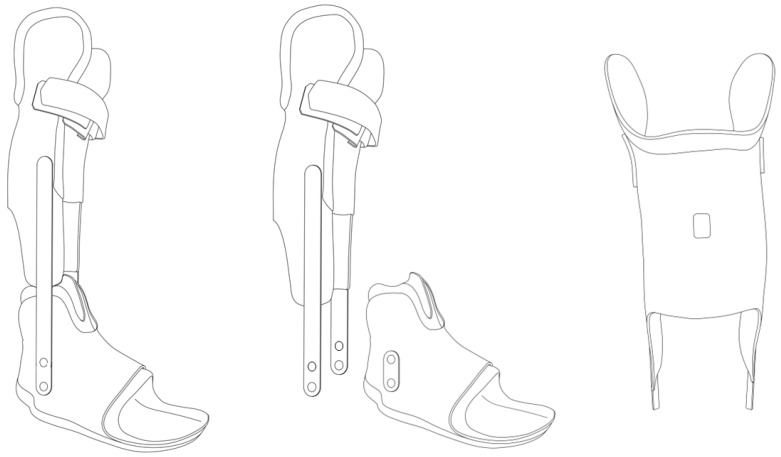
Schematic example of an orthosis for the Immobilization/Activity group (IAG) (**middle**) and Immobilization group (IG) (**left**). The orthoses were equipped with an Orthotimer sensor (**right**) (Orthotimer, Balingen, Germany). The IAG used the same type of orthosis as the IG; however, the lower leg shell was removable to allow calf muscle activity (**middle**). Permission obtained from the copyright holder (© Daniel Suda) to reproduce this figure in this paper.

The participants in the IG received a “golden standard” treatment, a dynamic ankle–foot orthosis (AFO, Figure 1) for night and day use for 23 h per day. The IAG participants were treated with the same type of AFO at night (8 h) and for 6 h during the day (altogether AFO treatment 14 h per day).

For the rest of the day (10 h), the participants used only the foot shell (FS) of the orthosis without the lower leg shell. The FS still secured the correct position of the foot and allowed free movement of the ankle joint with correction of the foot deformity (e.g., Pes equinovarus/equinovalgus, midfoot-break). At the local medical center, orthotic wear time of approximately 23 h/day is the standard approach to maximize passive stretch and mechanical alignment of the gastrocnemius–Achilles-tendon unit. To allow sufficient opportunities for active movement while maintaining mechanical stimulation, a reduced wearing schedule of 14 h/day was selected for the IAG. In line with the daytime wearing duration proposed by Tardieu et al. [25], approximately 6 h of orthosis use were maintained during the day, while the remaining wearing time (≈8 h) was scheduled during nighttime sleep. This approach allowed substantial daily mechanical stimulation while preserving extended periods of active daytime mobility. Both regimens, therefore, reflect clinically motivated treatment strategies rather than a formal dose–response study.

**Figure 2:**
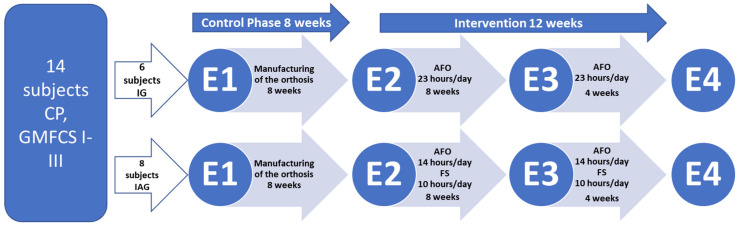
Timeline of the study. Fourteen ambulatory children and adolescents with spastic cerebral palsy (CP) and equinus deformity were randomized into two groups: an Immobilization Group (IG) and an Immobilization/Activity Group (IAG). The control phase lasted 8 weeks (E1–E2). The intervention phase lasted 12 weeks in total (E2–E4). E1: examination of muscle morphology, muscle function, and mechanics before manufacturing of the orthoses; E2: examination before the intervention; E3: examination after 8 weeks of intervention; E4: examination after 12 weeks of intervention. GMFCS: Gross Motor Function Classification System.

Before the start of the orthotic intervention, an 8-week control phase was conducted (Figure 2). During this time, individual orthoses for each participant had been manufactured. Afterwards, the 12-week orthotic intervention phase was performed. A 12-week intervention period was selected based on previous studies demonstrating that skeletal muscle structural adaptations to altered mechanical loading occur within several weeks. With regard to orthotic treatment, significant changes in GM fascicle length were reported after approximately 16 ± 4 weeks [19]. Therefore, a 12-week duration was considered sufficient to capture clinically relevant adaptations in muscle architecture while remaining feasible in a pediatric clinical setting.

Muscle morphology was examined four times (before the manufacturing of the orthosis (E1), at the beginning of the orthotic treatment (E2), after 8 weeks of orthotic wear (E3), and finally, after 12 weeks of orthotic treatment (E4) (Figure 2).

### 2.4. Orthotic Intervention

In both groups, the orthosis was set up such that plantarflexion of the foot was restricted, but dorsiflexion was unrestricted. The maximum plantarflexion setting was adjusted towards dorsiflexion approximately every third week, depending on clinical improvement. To verify whether the prescribed wearing time was met during the intervention, each orthosis was equipped with a specific temperature sensor (Orthotimer, Balingen, Germany). The sensor was attached to the hard shell of the orthosis and measured and recorded temperature every 15 min. The stored data was read out after the intervention using a wireless reader and transferred to a computer for further analysis. In addition, an activity diary was provided in which parents and caregivers recorded the days and times the orthosis was worn, as well as the activities performed during those times.

#### 2.4.1. Immobilization Group

Every participant of the IG was provided with an individually manufactured AFO (Figure 1). The aim of the orthosis application was not only to correct the equinus position at the ankle joint but also to control for midfoot break deformity at the Chopart and subtalar joints. A soft inner layer ensured that the foot was comfortable within the orthosis. A hard outer shell that was built through a laminar technique (matrix and several fiber composites) and used for both the lower leg and FS. An S-type calf-construction with condylar support was utilized as the lower leg shell. The FS used a circular foot support. The subtalar joint was locked by a circular frame, and the heel was fixed with a removable heel cap. Both parts (foot and lower-leg shells) were linked by a constrained metal ankle hinge aligned in maximum passive dorsiflexion while keeping the knee extended without discomfort.

#### 2.4.2. Immobilization/Activity Group

The innovative part of the IAG orthosis was the linkage between the FS and the lower-leg shell, which allowed the FS to be used independently of the lower-leg shell. This allowed the calf muscles to be immobilized and stretched to improve equinus and to control for foot deformities, without limiting ankle joint range of motion during the active part of the intervention regime. Due to this new feature, the plantarflexors should have been more active during the day in the IAG.

### 2.5. Data Collection and Processing

#### 2.5.1. Morphological and Architectural Muscle–Tendon Properties

The morphological properties of the GM muscle and the Achilles tendon were examined in the passive condition using three-dimensional (3D) freehand ultrasound, an already-validated measurement technique [26]. Participants were positioned prone on a bench with their feet extending beyond its edge. To enable controlled ankle rotation in the sagittal plane and to stabilize the subtalar joint, a custom-built footplate was secured to the foot; this device incorporated an inclinometer and a detachable torque wrench [26,27]. The neutral resting ankle angle was first identified, corresponding to the dorsiflexed joint position where no external torque (0 Nm) was applied. While the ankle was maintained in this resting position, a series of transverse ultrasound sweeps extending from the knee to the calcaneus was performed to assess muscle volume [26]. Additionally, a single transverse sweep was acquired to determine muscle architectural properties [26]. Both scan types were repeated twice, with the higher-quality dataset selected for further analysis. To ensure that tissue properties were measured in a passive condition, surface electromyography (EMG) of the tibialis anterior was recorded during the full sweeps using an EMG system (myon 320, myon AG, Zurich, Switzerland). During single sweeps, EMG signals were also collected from the gastrocnemius lateralis muscle. The EMG was sampled at 2000 Hz, with signal quality checked both during acquisition and in post-processing to confirm the absence of voluntary activation. Skin preparation and electrode placement (Blue Sensor N, Ambu A/S, Ballerup, Denmark) followed the SENIAM guidelines [28] and were further verified with ultrasound.

**Figure 3:**
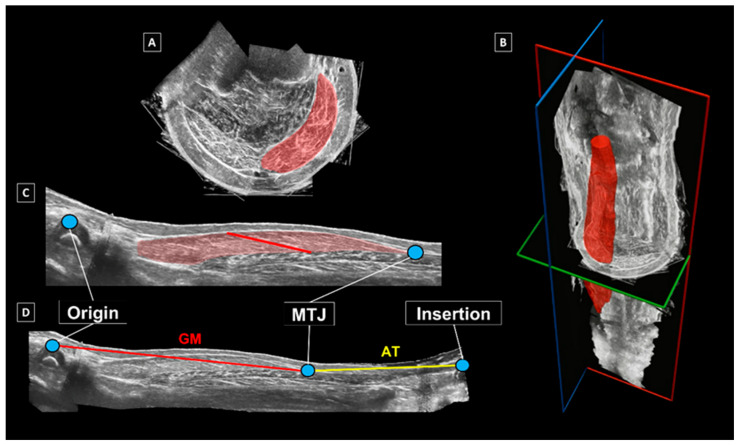
Outcomes of the 3D freehand ultrasound analysis. (**A**) Segmented boundaries of the gastrocnemius medialis (GM) muscle in the transverse plane; (**B**) segmented and interpolated 3D model of the GM muscle belly (red); (**C**) determination of GM fascicle length within the mid-longitudinal muscle plane; (**D**) assessment of GM muscle belly and Achilles tendon lengths [30].

A full 3D reconstruction of the GM muscle–tendon unit (MTU) was created by combining marker-cluster positional data with ultrasound scans in MATLAB (version 7.1, MathWorks Inc., 2005) [26]. From the reconstructed images obtained via the full sweeps, GM muscle volume was estimated by manually outlining its borders in transverse sections, beginning at its anatomical origin—defined by the medial and lateral femoral epicondyles—and ending at the muscle–tendon junction (MTJ). This segmentation was performed in the Medical Imaging Interaction Toolkit (MITK) (Figure 3A), with boundaries delineated at intervals of approximately 2 cm along the muscle length. A 3D representation of the GM was then produced by interpolating between segmented slices using ITK-SNAP [29] (Figure 3B). Muscle volume was determined as the sum of all segmented and interpolated voxels within the reconstructed model.

#### 2.5.2. Gastrocnemius Medialis Muscle Belly and Achilles Tendon Lengthening Behavior

**Figure 4:**
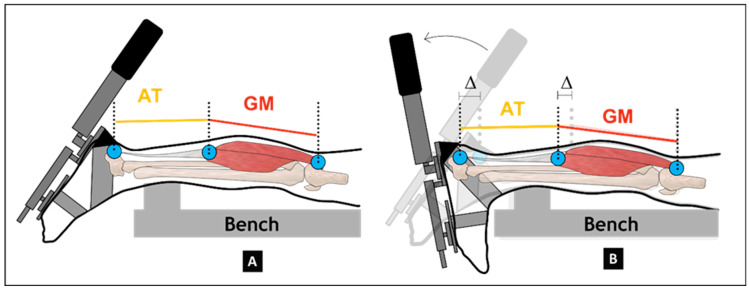
Dynamic measurements of the gastrocnemius muscle belly and Achilles tendon lengths during passive ankle dorsiflexion from maximum plantarflexion (**A**) to maximum dorsiflexion (**B**) within the sagittal plane [30].

A detailed description of the methodology for evaluating the dynamic lengthening behavior of the GM muscle belly and the Achilles tendon is available elsewhere [30]. In brief, after collecting the 3D freehand ultrasound data, reflective markers were fixated at the anatomical origin and insertion sites. In addition, a 59-mm linear-array ultrasound probe (LogicScan 128; Telemed, Vilnius, Lithuania) mounted with a rigid cluster of four reflective markers was positioned over the GM muscle–tendon junction (MTJ) to track its displacement. Muscle–tendon junction motion was sampled at a frequency of 60 Hz. To induce controlled ankle motion, the inclinodynamometer was used to continuously rotate the footplate from maximal plantarflexion (Figure 4A) to maximal dorsiflexion (Figure 4B) in the sagittal plane.

During the dorsiflexion movement in the sagittal plane, the external torque applied to the footplate, the lengths of the GM muscle belly and Achilles tendon, as well as the EMG activity of the tibialis anterior and gastrocnemius lateralis were recorded simultaneously. Each participant performed two dorsiflexion trials at a slow angular velocity (~20°/s), and mean values across the repetitions were used for statistical evaluation. In the post-processing phase, displacement of the MTJ was manually tracked in the ultrasound recordings using Tracker software (version 4.91) [31,32]. To derive accurate in vivo tissue lengths, the positions of the reflective markers were mathematically corrected to match the anatomical landmarks [30]. Muscle belly length was defined as the straight-line distance between the corrected anatomical origin and the MTJ, while tendon length was calculated as the distance from the MTJ to the corrected heel marker. The total MTU length was obtained as the sum of these two measures. Changes in GM muscle belly and Achilles tendon length were analyzed across a standardized torque interval of 0 Nm to 6 Nm, a range that all participants were able to complete without discomfort. Based on previous approaches, the tissue lengths and length changes were normalized to lower leg length measured from the lateral tibial plateau to the lateral malleolus (mm) [32,33].

### 2.6. Statistical Analysis

All statistical analyses were conducted using SPSS (version 26, SPSS Inc., Chicago, IL, USA). The significance level was set to *p* = 0.05. Data were tested for normality using the Shapiro–Wilk test, histograms, and Q-Q plots. Paired *t*-tests were performed to assess the difference between the measured wearing time and the time of the daily activity log. A linear mixed model with comparison of the main effects with Bonferroni correction was performed to assess the effects of the orthotic treatment. The within-subjects factor was time, with four levels: FAM (E1), PRE (E2), POST (E3), and FOLLOW-UP (E4) (Figure 2). The between-subjects factor was orthosis group, with two levels: IG and IAG. Linear mixed-effects models were used to analyze longitudinal changes, as they appropriately account for repeated measurements within subjects and allow inclusion of all available observations without requiring case-wise deletion, which represents a methodological advantage over traditional repeated-measures ANOVA in longitudinal clinical datasets. This statistical approach was selected to appropriately address the longitudinal study design and the research objectives. Furthermore, the minimal detectable change (MDC) in MTU length was calculated in MATLAB (The MathWorks Inc., Natick, MA, USA) using the standard error of measurement and the critical t-value obtained from paired t-tests, to estimate the daily orthosis wearing time required to induce a detectable physiological change. All available data were included in the analysis. Dropouts occurred only due to missed visits or incomplete data acquisition, caused by the lack of patient cooperation during the measurement. No other data were excluded. Missing data were handled within the linear mixed model framework, and corresponding details for each outcome parameter are reported in the Results section.

## 3. Results

Significant effects were found for the objectively measured wearing time between PRE-POST and POST-FOLLOW (*p* = 0.01; Cohen’s d = 0.77), and between the measured time and the self-assessed daily activity diary for PRE-POST (*p* = 0.014; Cohen’s d = 0.73) and POST-FOLLOW (*p* = 0.014; Cohen’s d = 0.73) (Table 2) for the pooled group.

No significant effect of time (*p* = 0.10, F(3, 28.95) = 2.25) and group × time interaction (*p* = 0.49) was observed for normalized GM muscle volume.

Furthermore, no significant effect of time (*p* = 0.08, F(3, 28) = 2.52) and group × time interaction (*p* = 0.48) was observed for normalized GM muscle belly length at 0 Nm and 4 Nm. At 4 Nm, normalized GM muscle belly length showed no significant effect for time (*p* = 0.36) and group × time interactions (*p* = 0.12) (Table 3).

However, AT length showed significant time effects at both 0 Nm (*p* = 0.01, F(3, 28.05) = 4.21) and 4 Nm (*p* = 0.01, F(3, 26.17) = 4.91), with increases in length of +1.21% and +1.99% observed in the IG and IAG between PRE and POST, respectively. No significant group or group × time interactions were found (*p* > 0.25).

Normalized MTU length demonstrated significant time effects, with increases at both 0 Nm (*p* < 0.001, F(3, 28.03) = 7.53, +1.05%) and 4 Nm (*p* < 0.001, F(3, 26.13, +0.19%) = 13.70). Again, no group × time interactions were observed (*p* = 0.70 and *p* = 0.27, respectively), indicating similar adaptations across IG and IAG. When analyzed by group, the MDC in MTU length corresponded to a minimum daily wearing time of 9.8 h/day for the IG and 11.8 h/day for the IAG, considering the measured wearing time of the lower leg shell between PRE and POST. FOLLOW was excluded from this analysis due to data incompleteness.

The ratio of GM to MTU length remained stable with no significant time effects (*p* = 0.35) or interactions (*p* = 0.28).

Regarding the lengthening behavior, neither the GM muscle belly, the AT, nor the MTU showed significant main effects (all *p* > 0.07) or interactions (all *p* > 0.99).

With regard to all other architectural GM muscle properties (fascicle length, muscle thickness, and pennation angle), no significant time effects (all *p* > 0.06) or interactions (all *p* > 0.10) were observed.

## 4. Discussion

This study investigated the effects of two different AFO approaches on the morphological and mechanical properties of the GM MTU in children and adolescents with spastic CP. During the 12-week intervention period, no significant changes in muscle size and architecture were observed in either group. However, significant increases in AT and MTU lengths occurred independently of group allocation. These results may suggest that orthotic-induced stretching primarily affects the tendon rather than the muscle tissue. Meanwhile, the addition of an activity-based component does not appear to provide measurable morphological benefits.

Recent international research highlights the importance of individualized orthotic management in children with neuromotor disorders. A recent systematic review and meta-analysis by Faccioli et al. [34] reported that AFOs can improve gait performance in children and adolescents with neuromotor disabilities, including those with cerebral palsy, particularly in stride parameters and ankle positioning. Importantly, the authors suggest that flexible orthotic designs may better preserve push-off power during gait compared to rigid AFOs. Similarly, a randomized trial of Bjornson et al. [35] demonstrated that individualized orthotic alignment and footwear design can significantly improve balance and parent-reported mobility after three months of intervention compared with non-individualized orthotic approaches.

These findings emphasize that orthotic treatment strategies should consider not only the orthosis type but also individual design parameters and patient-specific functional goals. In this context, optimizing orthosis management, including wearing schedules and orthotic design, may help balance the therapeutic management of muscle contractures with the preservation of muscle function, strength, and gait performance in children with CP.

In the present study, we did not find significant changes over time in the morphology and architecture of the GM muscles in our participants, indicating that the imposed stretch stimulus was likely insufficient to induce measurable longitudinal growth of the muscle fibers. This finding is consistent with earlier observations that passive stretching alone does not effectively stimulate fascicle elongation in spastic muscles [8,9]. Spastic muscles exhibit altered mechanical properties, including increased stiffness, reduced sarcomere adaptability, and limited responsiveness to lengthening stimuli [36]. Consequently, the static stretch applied by orthotic positioning may not provide the dynamic mechanical loading necessary to trigger structural remodeling of the contractile tissue, as also indicated by Hösl et al. [19]. Thus, in contrast to Hösl et al., both orthotic interventions applied in this study did not result in measurable GM muscle atrophy or architectural deterioration (i.e., reduced muscle belly length and fascicle shortening) of the respective muscle. We assume that the difference in intervention duration between the study by Hösl and colleagues (mean wearing time of 16 weeks) and our study (12 weeks) was the decisive factor. This observation could justify the safe clinical use of day and night orthoses for periods of no longer than three months, as opposed to the four months applied by Hösl et al. [19]. However, this assumption should be proven in future studies.

Nevertheless, in both groups, we observed significant increases in AT (+1.21%) and MTU length (+1.05%) during the intervention period, suggesting that structural adaptations occurred within the tendon rather than the muscle. This response is consistent with the known mechanobiological adaptability of tendinous tissue, which reacts to sustained mechanical tension by increasing its length and compliance (Maganaris et al., 2006 [37]; Kubo et al., 2012 [38]). The observed increase in AT length is likely driven by chronic tensile loading during orthotic dorsiflexion positioning, suggesting that tendon elongation is the primary mechanism underlying MTU lengthening. This finding is in line with previous results [19].

Maintaining the relative ratio between GM muscle belly and MTU lengths supports the hypothesis that the muscle itself remained structurally stable while the tendon adapted. From a functional perspective, such tendon elongation could facilitate improved ankle dorsiflexion range and may reduce the risk of equinus gait progression or secondary skeletal deformities, which are common in spastic CP [39,40]. However, further research is required to ascertain whether these morphological tendon changes result in quantifiable enhancements in joint kinematics or gait efficiency.

The introduction of an additional daily activity protocol in the IAG did not result in enhanced morphological adaptations in comparison to immobilization alone. This finding indicates that the level and intensity of voluntary muscle activation achieved within the intervention were inadequate to induce structural remodeling beyond that achieved by passive stretching. It has been demonstrated that active contractile loading, rather than sarcomere lengthening, is imperative for muscle growth and fascicle adaptation [8,39]. Limited voluntary muscle activation in the spastic GM may have limited the potential benefits of the IAG approach. Consequently, future interventions should consider integrating dynamic or resistance-based exercises, which have been shown to provide targeted, high-intensity activation of the plantarflexors [41]. This approach is hypothesized to enhance muscle adaptation potential.

The findings of the present study further suggest that a detectable physiological effect regarding changes in GM MTU length can be achieved over an 8-week period with a daily orthosis wearing time of 9.8–11.8 h, exceeding the 6 h/day proposed by Tardieu et al. (1988) [42], but well below the 23 h/day regimes often applied in practice [43]. This may suggest that effective intervention may be possible with shorter, more tolerable wearing schedules, potentially enhancing patient compliance. Nonetheless, further studies are needed to determine the optimal wearing duration across different intervention periods, as it may vary with the total intervention length and the magnitude of the physiological response over time.

From a clinical perspective, the findings of the study may suggest that a substantially reduced daily immobilization period of approximately 14 h, rather than the traditionally recommended 23 h, is sufficient to achieve comparable effects on muscle–tendon parameters. This challenges the long-held assumption that nearly continuous orthotic wear is necessary to elicit meaningful biomechanical adaptations. These results are particularly relevant in pediatric and adolescent populations, where adherence and comfort are central concerns, as they support a more pragmatic and patient-centered approach to prescribing immobilization duration without compromising therapeutic benefit. Furthermore, the present results indicate that prolonged orthotic use for up to 12 weeks appears to be safe in young people with CP. The absence of muscle atrophy or fascicle shortening may suggest that moderate-duration immobilization using appropriately designed orthoses does not compromise the structural integrity of the spastic GM muscle. This may encourage clinicians to maintain orthotic treatment regimens aimed at preventing contracture progression without increasing the risk of disuse-related muscle degeneration. Moreover, the observed tendon adaptations may enhance joint mobility and reduce the need for more invasive procedures, such as tendon-lengthening surgery or repeated botulinum toxin injections. However, this study focused only on the GM muscle; therefore, the results cannot be generalized to other muscles, such as the gastrocnemius lateralis or the soleus.

Several aspects should be considered when interpreting the results. The participants were recruited from a single pediatric orthopedic center, potentially restricting the generalizability of the findings to broader populations with different clinical practices or demographic characteristics. Although none of the participants had a Botulinum toxin A application within one year before study participation, four of the participants received injections to the calf muscles several years before they participated (mostly at the age of four to five years). To the best of our knowledge, there is yet no clear evidence that the effects of Botox treatment are still present several years after the injection. However, we cannot state with certainty that any effects remained in the participants. Nevertheless, we assume that any remaining effects did not influence our results, given the pre–post design of our study. In addition, measurement bias cannot be entirely excluded, as blinding of assessors was not feasible and some outcomes were sensitive to participant adherence and tolerance, resulting in incomplete data for certain parameters. Dropouts occurred due to missed visits or intolerance to the orthosis; these instances were carefully documented and included in the analyses where possible. Taken together, these factors should be considered when interpreting the results, and larger multi-center studies with standardized assessment protocols are warranted to confirm and extend the observed effects. Moreover, the proposed study concentrated exclusively on morphological parameters, excluding functional outcomes such as strength, spasticity levels, and gait performance. The observed association between daily wearing time and physiological adaptation should be considered exploratory. As wearing time was not independently randomized and the study was not designed as a dose–response trial, no causal inferences or definitive recommendations regarding optimal wearing duration can be derived. Although the study had sufficient power based on the a priori sample size calculation, we used a small sample (*n* = 14), which may have constrained the statistical power to discern subtle group differences or interactions. Because of the natural variability among individuals with cerebral palsy, the generalizability of the study to the whole population should be interpreted with caution. The variability in participant age and biological growth status may have further influenced muscle and tendon development during the study period. Finally, due to limited personnel resources, blinding the examiners to group allocation was not possible in this study. We recommend that future studies on this topic use larger cohorts and longer follow-up periods to determine whether the observed tendon elongation is persistent and whether muscle adaptation occurs over extended periods. Furthermore, refining the activity component to encompass targeted, resistance-based, or neuromuscularly challenging exercises may facilitate the identification of thresholds necessary to induce meaningful changes in spastic muscle architecture.

## 5. Conclusions

In conclusion, a 12-week period of orthotic immobilization, with or without additional daily muscle activation, did not alter the morphology of the GM muscle of children and adolescents diagnosed with spastic CP. Conversely, structural adaptations were observed exclusively in the Achilles tendon, suggesting that tendon tissue is more responsive to orthotic-induced stretching than muscle tissue. The findings also indicate that partial-day orthosis use (approximately 14 h/day) can elicit meaningful muscular adaptations while potentially enhancing adherence, tolerance, and overall usability, balancing therapeutic effectiveness with patient adherence and tolerance.

The findings may also point to the relative resistance of spastic muscle architecture to low-level mechanical stimuli, while demonstrating that moderate-duration orthotic treatment does not appear to adversely affect muscle-tendon structure. These insights may contribute to optimizing conservative management strategies to preserve muscle-tendon function and prevent contracture progression in individuals with spastic CP. These results may support a patient-centered approach in pediatric orthotic management, allowing individualized wearing schedules that optimize both therapeutic effect and quality of life. In addition, increased active time during the day may help counteract immobilization-induced muscle atrophy by preserving opportunities for voluntary muscle activation and functional movement.

## Figures and Tables

**Table 1:** Participant characteristics of the children and adolescents with spastic cerebral palsy of the Immobilization Group (IG) and the Immobilization/Activity Group (IAG). Data is presented as mean (SD).

	IG	IAG
Number	6	8
Gender (female/male)	2/4	2/6
Age (years)	9.0 (2.7)	10.6 (2.9)
USCP/BSCP	3/3	5/3
Body mass (kg)	25.7 (5.0)	45.8 (17.0)
Body height (cm)	130.7 (12.5)	147.5 (16.3)
Lower leg length (cm)	30.0 (3.0)	34.3 (3.4)
GMFCS Level I/II	3/3	6/2
SCALE (points: 2/1/0)	0/6/0	1/7/0

Grading of the “Selective Control Assessment of the Lower Extremity” (SCALE) is presented for the more affected leg or the affected leg in children with bilateral spastic cerebral palsy (BSCP) and those with unilateral spastic cerebral palsy (USCP), respectively. Grading was limited to the ankle joint: normal: 2 points; impaired: 1 point; unable: 0 points.

**Table 2:** Wearing time of the orthoses, measured by a sensor (Orthotimer, Balingen, Germany) and documented in a daily activity log completed by the parents. Data are presented as mean (SD).

	Orthotimer			Daily Activity Log			Diff Orthotimer–Activity Log	
	Pre-Post	Post-Follow	Δ Diff	Pre-Post	Post-Follow	Δ Diff	Pre-Post	Post-Follow
Lower Leg Shell [h]	7.83 (4.81)	5.73 (6.29)	−2.1	9.92 (4.88)	8.76 (7.23)	−1.16	−21.07%	−34.59%
Foot Shell [h]	8.31 (3.08)	4.92 (4.44)	−3.39	15.09 (3.47)	11.93 (6.86)	−3.16	−44.94%	−58.76%

**Table 3:** Morphological and architectural gastrocnemius medialis muscle–tendon properties in children and adolescents with spastic cerebral palsy. Muscle volume was normalized to body mass. The tissue lengths and length changes were normalized to lower leg length measured from the lateral tibial plateau to the lateral malleolus (mm). The number of datasets used for each parameter is stated (number/available datasets). Data are presented as mean (SD).

Parameter	Effect	Group	FAM	PRE	POST	FOLLOW	DoF	F	Sig.
Muscle–tendon lengths and lengthening									
Norm. GM muscle length_0 Nm (56/64)	Time	IG	5.85 (0.31)	5.97 (0.31)	6.04 (0.31)	6.05 (0.31)	28.00	2.52	0.08
	Time X Group	IAG	5.74 (0.24)	5.80 (0.24)	5.78 (0.24)	5.81 (0.24)	28.00	0.85	0.48
Norm. AT length_0 Nm (56/64)	Time	IG	4.53 (0.22)	4.46 (0.22)	4.51 (0.22)	4.62 (0.22)	28.05	4.21	**0.01**
	Time X Group	IAG	4.46 (0.18)	4.48 (0.17)	4.60 (0.17)	4.59 (0.18)	28.05	1.40	0.26
Norm. MTU length_0 Nm (56/64)	Time	IG	10.38 (0.22)	10.44 (0.22)	10.55 (0.22)	10.67 (0.22)	28.03	7.53	**0.00**
	Time X Group	IAG	10.20 (0.17)	10.28 (0.17)	10.38 (0.17)	10.39 (0.17)	28.03	0.48	0.70
Ratio GM-MTU length_0 Nm [%] (56/64)	Time	IG	56.36 (2.29)	57.25 (2.29)	57.25 (2.29)	56.63 (2.29)	28.01	1.15	0.35
	Time X Group	IAG	56.13 (1.82)	56.33 (1.81)	55.57 (1.82)	55.78 (1.82)	28.01	1.33	0.28
Norm. GM muscle length_4 Nm (54/64)	Time	IG	5.94 (0.34)	6.22 (0.34)	6.15 (0.34)	6.17 (0.34)	26.05	1.12	0.36
	Time X Group	IAG	5.99 (0.27)	5.93 (0.27)	6.00 (0.27)	5.96 (0.27)	26.05	2.18	0.12
Norm. AT length_4 Nm (54/64)	Time	IG	4.49 (0.22)	4.53 (0.22)	4.62 (0.22)	4.73 (0.22)	26.17	4.91	**0.01**
	Time X Group	IAG	4.37 (0.18)	4.56 (0.18)	4.51 (0.18)	4.69 (0.18)	26.17	0.57	0.64
Norm. MTU length_4 Nm (54/64)	Time	IG	10.44 (0.25)	10.75 (0.25)	10.77 (0.25)	10.91 (0.25)	26.13	13.70	**0.00**
	Time X Group	IAG	10.36 (0.20)	10.49 (0.20)	10.50 (0.20)	10.64 (0.20)	26.13	1.37	0.27
Norm. GM muscle lengthening (40/64)	Time	IG	0.26 (0.08)	0.24 (0.10)	0.32 (0.08)	0.31 (0.08)	11.69	0.55	0.66
	Time X Group	IAG	0.25 (0.64)	0.23 (0.07)	0.26 (0.06)	0.22 (0.06)	11.69	0.50	0.69
Norm. AT lengthening (40/64)	Time	IG	0.11 (0.04)	0.02 (0.06)	0.06 (0.04)	0.05 (0.04)	13.82	1.90	0.18
	Time X Group	IAG	0.15 (0.03)	0.07 (0.04)	0.08 (0.03)	0.11 (0.03)	13.82	0.24	0.87
Norm. MTU lengthening (40/64)	Time	IG	0.37 (0.07)	0.24 (0.09)	0.39 (0.07)	0.35 (0.07)	11.40	2.84	0.09
	Time X Group	IAG	0.38 (0.06)	0.29 (0.06)	0.34 (0.06)	0.32 (0.06)	11.40	0.73	0.56
Muscle–tendon morphological properties									
Norm. fascicle length_0 Nm (56/64)	Time	IG	1.17 (0.13)	1.21 (0.13)	1.13 (0.13)	1.18 (0.13)	28.60	0.77	0.52
	Time X Group	IAG	1.24 (0.11)	1.35 (0.10)	1.22 (0.10)	1.19 (0.11)	28.60	0.19	0.90
Norm. muscle thickness_0 Nm (56/64)	Time	IG	0.38 (0.03)	0.38 (0.03)	0.39 (0.03)	0.40 (0.03)	28.11	0.52	0.67
	Time X Group	IAG	0.46 (0.02)	0.48 (0.02)	0.49 (0.02)	0.46 (0.02)	28.11	1.01	0.40
Norm. fascicle length_4 Nm (54/64)	Time	IG	1.39 (0.17)	1.49 (0.17)	1.32 (0.17)	1.22 (0.17)	26.58	1.80	0.17
	Time X Group	IAG	1.53 (0.14)	1.57 (0.14)	1.42 (0.15)	1.39 (0.15)	26.58	0.08	0.97
Norm. muscle thickness_4 Nm (54/64)	Time	IG	0.39 (0.02)	0.39 (0.02)	0.38 (0.02)	0.40 (0.02)	24.68	0.36	0.78
	Time X Group	IAG	0.46 (0.02)	0.48 (0.02)	0.48 (0.02)	0.44 (0.02)	24.68	2.30	0.10
Pennation angle_0 Nm [°] (56/64)	Time	IG	−4.41 (7.18)	−4.42 (7.18)	−3.55 (7.18)	−12.23 (7.18)	28.76	2.11	0.12
	Time X Group	IAG	−11.08 (6.21)	−14.63 (5.68)	−24.83 (5.93)	−24.79 (6.22)	28.76	0.91	0.45
Pennation angle_4 Nm [°] (54/64)	Time	IG	−4.51 (6.58)	−2.34 (6.58)	−10.82 (6.58)	−10.72 (6.58)	27.80	2.71	0.06
	Time X Group	IAG	−5.55 (5.48)	−10.92 (5.52)	−20.62 (5.88)	−21.97 (6.34)	27.80	0.41	0.75
Norm. fascicle lengthening (52/64)	Time	IG	0.19 (0.04)	0.28 (0.04)	0.21 (0.04)	0.19 (0.04)	25.71	0.61	0.61
	Time X Group	IAG	0.16 (0.03)	0.12 (0.04)	0.13 (0.04)	0.14 (0.14)	25.71	1.76	0.18
Norm. GM muscle volume [mL/kg] (57/64)	Time	IG	2.13 (0.35)	2.18 (0.35)	2.27 (0.35)	2.26 (0.35)	28.95	2.25	0.10
	Time X Group	IAG	2.11 (0.28)	2.39 (0.28)	2.33 (0.28)	2.29 (0.28)	28.95	0.83	0.49

GM: gastrocnemius medialis; AT: Achilles tendon; MTU: muscle–tendon unit; Norm: normalized; IG: Immobilization Group; IAG: Immobilization Activity Group; DoF: degrees of freedom. Bold numbers: significant difference (*p* < 0.05).

## Data Availability

Data can be made available on reasonable request.

## Abbreviations

The following abbreviations are used in this manuscript:

AFO	Ankle–foot orthosis
AT	Achilles tendon
CP	Cerebral palsy
EMG	Electromyography
FS	Foot shell
GM	Gastrocnemius medialis
GMFCS	Gross motor function classification system
IG	Immobilization group
IAG	Immobilization/Activity group
MDC	Minimal detectable change
MTJ	Muscle-tendon junction
MTU	Muscle-tendon unit
SCALE	Selective Control Assessment of the Lower Extremity
